# Anxiety prevalence and associated factors among frontline nurses following the COVID-19 pandemic: a large-scale cross-sectional study

**DOI:** 10.3389/fpubh.2023.1323303

**Published:** 2023-12-07

**Authors:** Shitao Wang, Guoshuai Luo, Dongsheng Pan, XiangQian Ding, Fei Yang, Liping Zhu, Shuo Wang, Xuelu Ma

**Affiliations:** ^1^Department of Neurology, Affiliated Fuyang People's Hospital of Anhui Medical University, Fuyang, China; ^2^Laboratory of Biological Psychiatry, Institute of Mental Health, Tianjin Anding Hospital, Mental Health Center of Tianjin Medical University, Tianjin, China; ^3^Department of Clinical Medicine, Anhui Medical University, Hefei, China; ^4^Department of Neurosurgery, Qilu Hospital of Shandong University, Jinan, China; ^5^Department of Neurology, Affiliated Hospital of North Sichuan Medical College, Nanchong, China; ^6^Ya'an People's Hospital, Sichuan University, Yaan, China

**Keywords:** anxiety symptoms, clinical nurses, interventions, post-pandemic period, related factors

## Abstract

**Introduction:**

Nurses are more likely to experience anxiety following the coronavirus 2019 epidemic. Anxiety could compromise nurses’ work efficiency and diminish their professional commitment. This study aims to investigate nurses’ anxiety prevalence and related factors following the pandemic in multiple hospitals across China.

**Methods:**

An online survey was conducted from April 16 to July 3, 2023, targeting frontline nurses who had actively participated in China. Anxiety and depression symptoms were assessed using the Self-rating Anxiety Scale and the Self-rating Depression Scale (SDS), respectively. Multivariable logistic regression analysis was employed to identify factors linked with anxiety.

**Results:**

A total of 2,210 frontline nurses participated in the study. Overall, 65.07% of participants displayed clinically significant anxiety symptoms. Multivariable logistic regression revealed that nurses living with their families [2.52(95% CI: 1.68–3.77)] and those with higher SDS scores [1.26(95% CI: 1.24–1.29)] faced an elevated risk of anxiety. Conversely, female nurses [0.02(95% CI: 0.00–0.90)] and those who had recovered from infection [0.05(95%CI: 0.07–0.18)] demonstrated lower rates of anxiety.

**Discussion:**

This study highlights the association between SDS score, gender, virus infection, living arrangements and anxiety. Frontline nurses need to be provided with emotional support to prevent anxiety. These insights can guide interventions to protect the mental well-being of frontline nurses in the post-pandemic period.

## Introduction

During the coronavirus 2019 (COVID-19) pandemic, occupational health issues have escalated rapidly in workplaces (1). Extended use of personal protective equipment has been linked to an increase in dermatological reactions (2). From December 2019 to June 2020, a significant number of frontline healthcare workers, totaling 22,380, who were caring for COVID-19 patients, reported experiencing anxiety, depression, or stress (3). Therefore, the adoption of suitable coping styles may be pivotal in mitigating the negative impacts on mental health (4).

Nurses occupy a pivotal role in managing the COVID-19 pandemic (5). Their continuous engagement in combating the pandemic, often marked by extended work hours and minimal rest opportunities, has rendered them susceptible to psychological distress (6). The incidence of mental health problems in nurses is higher than that of the general population due to an array of stressors (7). Emerging evidence suggests that the psychological ramifications of the pandemic are enduring (8–12).

It is very common for clinical nurses to experience anxiety and depression (13). Notably, their anxiety levels throughout the pandemic exceeded other healthcare workers (14). Approximately 37% clinical nurses reported anxiety during the breakout of the pandemic (15). They were still troubled by anxiety even in the late stage of the epidemic (16). Excessive working hours, fear of infection, decision-making dilemmas in care prioritization, and shortages of equipment were identified as primary anxiety sources among nurses (17, 18). Severe anxiety could compromise their work efficiency and diminish their professional commitment (19). An integrated approach to managing the factors that impact the mental health of frontline nurses can effectively alleviate their psychological distress, thereby enhancing the quality of care provided (20). However, to our knowledge, no previous study specifically assessed anxiety and associated factors among Chinese nurses following the COVID-19 pandemic. Thus, comprehending anxiety’s determinants among nurses holds potential for guiding anxiety-alleviating strategies.

We conducted the present study to evaluate the mental health outcomes among a large sample of Chinese nurses following the COVID-19 pandemic by evaluating anxiety symptoms, and by analyzing associated risk factors.

## Materials and methods

### Study design

This is a large-scale cross-sectional and multicentre study that investigated clinical nurses’ anxiety symptoms and its associated factors following the COVID-19 epidemic. The description of the study was done following STROBE guidelines for reporting observational studies (21).

### Setting and participants

A large-scale online survey was performed to collect data from frontline nurses following the COVID-19 pandemic in 27 provinces in China. Raosoft^1^ was used to assess the sample size required for this study. According to China Daily,^2^ the total number of nurses has now exceeded 5.2 million. A minimum of 385 nurses was needed with margin of error (5%), confidence level (95%) and response distribution (50%).

Inclusion criteria: (1) Nurses engaged in frontline work following the COVID-19 pandemic, and (2) voluntary participation in this study. Exclusion criteria: (1) Lack of the professional qualification certificate, and (2) unwilling to participate in this study.

### Assessment measures

Demographic characteristics (title, gender, age, personality, virus infection, economic pressure, living style, worried about being infected, and SDS score) were collected. Anxiety was assessed via the Self-rating Anxiety Scale (SAS) (22). The Zung SAS contains 20 items, each with a 4-point scale ranging from 1 (none) to 4 (all of the time). A cut-off score of 36 was used to screen for clinically significant anxiety symptoms (23). Depression levels were assessed using the Self-rating Depression Scale (SDS) (24), which consists of 20 items. Each item on the scale is rated from 1 to 4. The total score is computed using the standard scoring method, where the SDS score is multiplied by 1.25. In the Chinese population, the Cronbach’s alpha values for the SAS and SDS were determined to be 0.92 and 0.93, respectively, demonstrating high reliability (25).

### Statistical analysis

SPSS 23.0 was used for Statistical analyses. Categorical variables were presented as frequencies and percentages. Comparisons between categorical variables were performed by chi square (χ^2^) test, and SDS scores between anxious and non-anxious participants were compared by the independent t test. Multivariate regression analysis was used to evaluate the impact of the diverse factors with statistically significant differences in χ^2^ or t-test on anxiety. The results of multivariate regression analysis were shown as adjusted odds ratios (ORs) and 95% confidence intervals (95%CIs). A *p* was considered significant if <0.05. For Bonferroni correction, a *p* < 0.001 (0.01/10) was considered significant. In continuous variables, receiver operating characteristic (ROC) analysis was used to assessed associated variable predictive value for anxiety. GraphPad Prism 9.0 generated forest plots.

## Results

### Demographic characteristics

Ultimately, a total of 2,210 nurses were included in the study based on inclusion and exclusion criteria. Most nurses were between 26 and 35 years old. Of the participants, 80.27% were women, and 68.14% lived with their families. More details of the participants are shown in Table 1. There were statistically significant differences between anxious and non-anxious participants in terms of SDS score, gender, virus infection and living style after Bonferroni correction (*p* < 0.001) (Tables 2, 3).

**Table 1:** General demographic characteristics of nursing staffs.

Demographic characteristic	*N*	%
Title		
Nurse (and below)	523	23.67
Nurse practitioner	774	35.02
Nurse-in-charge (and above)	913	41.31
Gender		
Male	436	19.73
Female	1774	80.27
Employment status		
Permanent employment	686	31.04
Temporary employment	1,524	68.96
Age (years)		
≤ 25	379	17.15
26 ~ 35	1,149	51.99
36 ~ 45	488	22.08
> 45	194	8.78
Personality		
Introvert	1,159	52.44
Extroversion	1,051	47.56
Virus infection		
Under infection	134	6.06
Recovery	1863	84.29
No infection	213	9.64
Economic pressure		
Yes	1,614	73.03
No	596	26.97
Living style		
Living alone	424	19.19
Living with family	1,506	68.14
Living with colleagues	280	12.67
Worried about being infected		
Yes	1,116	50.50
No	1,094	49.50

**Table 2:** Comparison of general demographic characteristics of nursing staffs.

Demographic characteristic	SAS score		χ^2^	*p*
	Anxiety (SAS⩾36)	Non-anxiety (SAS < 36)		
Title			10.70	*
Nurse (and below)	333 (15.07%)	190 (8.60%)		
Nurse practitioner	538 (24.34%)	236 (10.68%)		
Nurse-in-charge (and above)	567 (25.66%)	346 (15.66%)		
Gender			22.50	**
Male	326 (14.75%)	110 (4.98%)		
Female	1,112 (50.32%)	662 (29.95%)		
Employment status			5.20	0.02
Permanent employment	470 (21.27%)	216 (9.77%)		
Temporary employment	968 (43.80%)	556 (25.16%)		
Age (years)			4.17	0.24
≤ 25	258 (11.67%)	121 (5.48%)		
26 ~ 35	750 (33.94%)	399 (18.05%)		
36 ~ 45	301 (13.62%)	187 (8.46%)		
> 45	129 (5.84%)	65 (2.94%)		
Personality			4.17	0.04
Introvert	777 (35.16%)	382 (17.29%)		
Extroversion	661 (29.91%)	390 (17.65%)		
Virus infection			81.18	**
Under infection	128 (5.79%)	6 (0.27%)		
Recovery	1,144 (51.76%)	719 (32.53%)		
Non infection	166 (7.51%)	47 (2.13%)		
Economic pressure			9.59	*
Yes	1,081 (48.91%)	533 (24.12%)		
No	357 (16.15)	239 (10.81%)		
Living style			46.47	**
Living alone	315 (14.25%)	109 (4.93%)		
Living with family	909 (41.13%)	597 (27.01%)		
Living with colleagues	214 (9.68%)	66 (2.99%)		
Worried about being infected			8.24	*
Yes	694 (31.40%)	422 (19.10%)		
No	744 (33.67%)	350 (15.84%)		

SAS, Self-rating Anxiety Scale. The symbol * and ** indicate a significant difference (*p* < 0.01 and *p* < 0.001, respectively) between anxiety and non-anxiety.

**Table 3:** Comparison of SDS scores between anxious and non-anxious groups.

Variables	SDS score		*t*	*p*
	Mean	SD		
Anxiety or non-anxiety			−41.21	*
Anxiety	60.84	8.59		
Non-anxiety	42.15	10.92		

SD, Standard Deviation; SDS, Self-rating depression scale. The symbol * indicates a significant difference (*p* < 0.001) between anxiety and non-anxiety.

### Risk factors of anxiety

**Figure 1:**
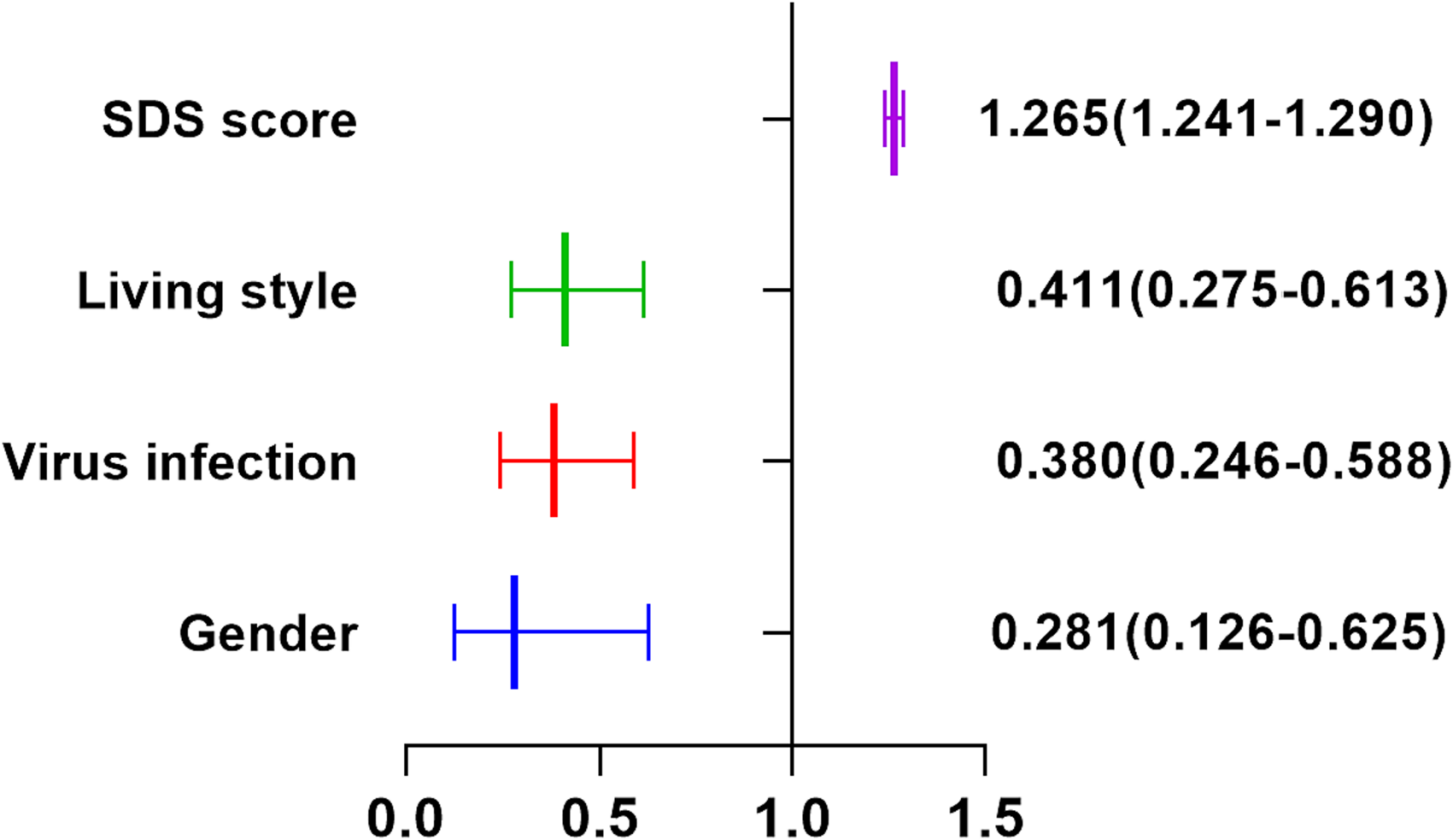
Forest plot for logistic regression analysis of the influencing factors of anxiety among nursing staffs.

We further evaluated the relationship between anxiety and factors with statistically significant differences in Tables 2, 3 using multivariate regression analysis. We found an association between SDS score, gender, virus infection, living style and anxiety (Figure 1).

As shown in Table 4, multivariable logistic regression analysis was performed for anxiety (SAS⩾36) by categorical covariates. Overall, nurses who lived with their families or had higher SDS scores had higher percentages of anxiety, while female nurses or nurses who had recovered from infection had lower percentages of anxiety.

**Table 4:** Multivariable logistic regression analysis for anxiety (SAS⩾36) by categorical covariates.

Variables	Adjusted OR (95% CI)	Category *p* value	Overall *p* value
**Gender**			
Male	1		*
Female	0.02 (0.00–0.90)	*	
**Virus infection**			
Non infection	1		*
Under infection	2.46 (0.79–7.67)	0.12	
Recovery	0.05 (0.07–0.18)	*	
**Living style**			
Living alone	1		*
Living with family	20.00 (0.43–935.81)	0.13	
Living with colleagues	7.94 (0.17–378.84)	0.29	
Living with colleagues	1		
Living with family	2.52 (1.68–3.77)	*	
**SDS score**	1.26 (1.24–1.29)	–	*

SAS, Self-rating Anxiety Scale; SDS, Self-rating depression scale. The symbol * indicates a significant difference (*p* < 0.05).

**Figure 2:**
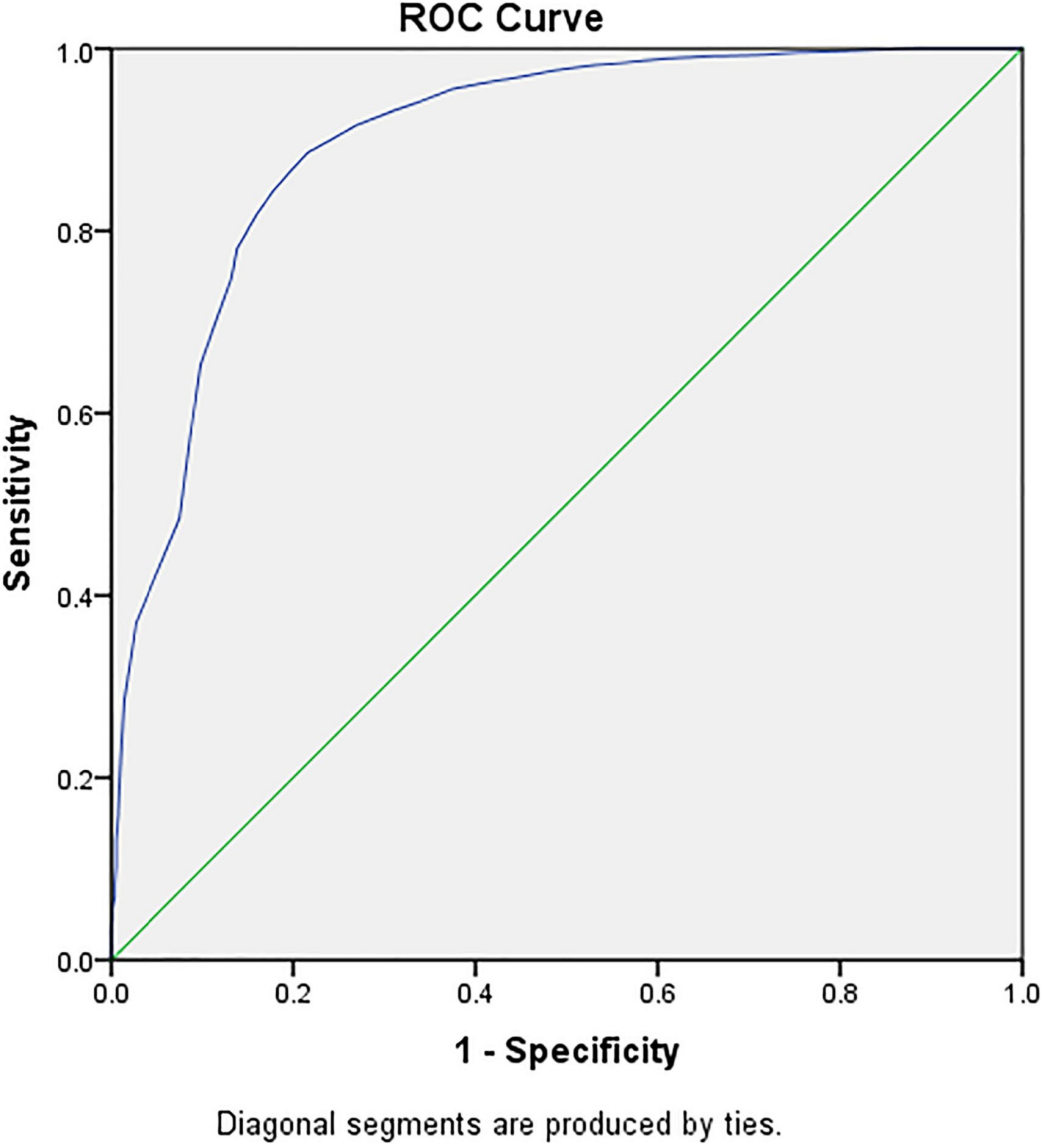
ROC analysis of SDS score influencing anxiety. Area under ROC curve is 0.898.

ROC analysis indicated SDS score has a good predictive value for anxiety (AUC = 0.90, 95% CI: 0.88–0.91) (Figure 2).

## Discussion

This study underscored that a significant proportion of frontline nurses experienced anxiety following the COVID-19 pandemic (65.07%), slightly surpassing the proportion observed in China’s later pandemic stages (54%) (16). Potential reasons for this inconsistency may be associated with the difference in methods as this study (16) adopted the different cut-off scores and different assessment scales to define clinically significant anxiety. However, a validated cut-off score was used to detect anxiety symptoms in our study. Moreover, sporadic COVID-19 cases in China post-pandemic might contribute to sustained frontline nurse anxiety levels.

Our result is consistent with that of a previous study, as the prevalence of anxiety among nurses during the COVID-19 pandemic was 71.9% (26), which might be because the epidemic in Turkey was already under control when Gül et al. conducted his study (26). However, the proportion of participants with clinically significant anxiety symptoms is slightly smaller than that in Turkey. It is possible that nurses in Turkey suffered from more anxiety since they have never experienced such a serious epidemic in the past, or even learned the knowledge of controlling the epidemic. Compared with nurses in Turkey, Chinese nurses were more confident in managing the epidemic based on their previous experience.

Female participants reported lower anxiety than male participants, probably because the latter were less confident and more uncertain about the future than the former. However, a recent study has discovered that female participants are more prone to experiencing stress and anxiety (27). The discrepancy in the survey results may be associated with the fact that our study only included nurses who worked in hospitals following the epidemic. The new findings provided important evidence for managers to formulate new psychological intervention strategies in male nurses following the epidemic.

Our results showed that participants who have recovered from the COVID-19 infection following the epidemic have a lower risk of anxiety compared to those who have not been infected. The possible reasons are explained as follows: First, they believed that they had developed antibodies due to previous infection and would be protected from being reinfected in the near future; Secondly, the clinical symptoms caused by the infection are not as serious as expected; Finally, they believed that there are already effective treatment methods and vaccines that can effectively prevent reinfection.

Participants who lived with their families experienced more anxiety than those living with colleagues because they not only need to do more housework, but also need to learn how to educate children, and take care of the older adult. The increased workload is associated with the risk of developing anxiety (28, 29). On the other hand, they did not have enough time for self-care. Research shows that self-care is very important for nurses (30). If nurses cannot maintain self-care, it will lead to stress, anxiety and burnout (31–33). Although research showed that social support is important for mental health (34), people who have been married are more likely to experience anxiety than those who are single (35).

We found that the higher the SDS score, the more likely the participants were to suffer from anxiety. This finding could be explained by the fact that anxiety and depression may share a common pathogenesis, or one disorder is an epiphenomenon of the other (36). According to our results of ROC analysis, SDS score had a good predictive value for anxiety elevation. However, the predictive potential of SDS score for anxiety elevation still needs to be further verified by studies in different population following the COVID-19 pandemic.

This study had some strengths and limitations. The strengths of this study: (1) Our study only included frontline nurses, which helps to understand the mental health outcome of frontline nurses following the COVID-19 pandemic and assists managers in formulating interventions and preventive actions, (2) Our study included frontline nurses from 27 provinces, and the findings are representative of the mental health outcome of frontline nurses following the epidemic, (3) Multidimensional examination of factors influencing anxiety; The possible limitations of this study: (1) Cross-Sectional Design: The cross-sectional design of this study restricts our ability to infer causal relationships between anxiety and the identified factors. Longitudinal studies are needed to better understand the temporal dynamics of anxiety among frontline nurses. (2) Self-Report Measures: The reliance on self-reported measures for anxiety and depression symptoms may introduce response bias and subjective interpretations. Objective clinical assessments could enhance the accuracy of mental health evaluations. (3) Social Desirability Bias: Participants might have provided responses they deemed socially desirable, potentially influencing the reported prevalence of anxiety or other associated factors. (4) Regional Focus: The study was conducted exclusively in China and might not fully represent the experiences and mental health outcomes of frontline nurses in other countries or cultural contexts. (5) Generalizability of Findings: While efforts were made to include a diverse sample from multiple provinces, the variation in healthcare settings, COVID-19 impact, and socioeconomic factors across regions might limit the generalizability of the study’s findings.

## Conclusion

In conclusion, the current study indicated an association between SDS score, gender, virus infection, living style and anxiety among frontline nurses, which may hopefully assist managers in implementing interventions following the COVID-19 pandemic.

## Data availability statement

The original contributions presented in the study are included in the article/supplementary material, further inquiries can be directed to the corresponding author.

## Ethics statement

The studies involving humans were approved by Ethics Committee of Tianjin Anding Hospital. The studies were conducted in accordance with the local legislation and institutional requirements. The participants provided their written informed consent to participate in this study.

## Author contributions

ShiW: Conceptualization, Funding acquisition, Methodology, Project administration, Resources, Writing – original draft. GL: Conceptualization, Data curation, Formal analysis, Software, Supervision, Validation, Writing – original draft. DP: Data curation, Writing – review & editing, Investigation, Supervision, Validation, Visualization. XD: Writing – review & editing, Data curation. FY: Writing – review & editing, Investigation, Resources. LZ: Formal analysis, Writing – review & editing, Data curation. ShuoW: Investigation, Writing – review & editing, Formal analysis, Software. XM: Investigation, Resources, Writing – review & editing.

## Footnotes

^1^http://www.raosoft.com/samplesize.html

^2^http://ex.chinadaily.com.cn/exchange/partners/70/rss/channel/www/columns/y38633/stories/WS64643210a310b6054fad35ef.html

## References

[1] Gualano MR Santoro PE Borrelli I Rossi MF Amantea C Daniele A. TElewoRk-RelAted stress (TERRA), psychological and physical strain of working from home during the COVID-19 pandemic: a systematic review. Workplace Health Saf. (2023) 71:58–67. doi: 10.1177/21650799221119155 36382962 PMC9672980

[2] Santoro PE Borrelli I Gualano MR Proietti I Skroza N Rossi MF. The dermatological effects and occupational impacts of personal protective equipment on a large sample of healthcare workers during the COVID-19 pandemic. Front Public Health. (2022) 9:815415. doi: 10.3389/fpubh.2021.815415 35141194 PMC8818717

[3] Salari N Khazaie H Hosseinian-Far A Khaledi-Paveh B Kazeminia M Mohammadi M. The prevalence of stress, anxiety and depression within front-line healthcare workers caring for COVID-19 patients: a systematic review and meta-regression. Hum Resour Health. (2020) 18:100. doi: 10.1186/s12960-020-00544-1 33334335 PMC7745176

[4] Rossi MF Gualano MR Magnavita N Moscato U Santoro PE Borrelli I. Coping with burnout and the impact of the COVID-19 pandemic on workers' mental health: a systematic review. Front Psych. (2023) 14:1139260. doi: 10.3389/fpsyt.2023.1139260 PMC10060559 37009102

[5] Shechter A Diaz F Moise N Anstey DE Ye S Agarwal S. Psychological distress, coping behaviors, and preferences for support among New York healthcare workers during the COVID-19 pandemic. Gen Hosp Psychiatry. (2020) 66:1–8. doi: 10.1016/j.genhosppsych.2020.06.007 32590254 PMC7297159

[6] Nie A Su X Zhang S Guan W Li J. Psychological impact of COVID-19 outbreak on frontline nurses: a cross-sectional survey study. J Clin Nurs. (2020) 29:4217–26. doi: 10.1111/jocn.15454 32786150 PMC7436701

[7] World Health Organization. Nursing and midwifery. Available at: https://www.who.int/news-room/fact-sheets/detail/nursing-and-midwifery

[8] Ausín B González-Sanguino C Castellanos MA Sáiz J Zamorano S Vaquero C. The psychological impact of the COVID-19 pandemic in Spain: a longitudinal study. Psicothema. (2022) 34:66–73. doi: 10.7334/psicothema2021.290 35048897

[9] Dharra S Kumar R. Promoting mental health of nurses during the coronavirus pandemic: will the rapid deployment of Nurses' training programs during COVID-19 improve self-efficacy and reduce anxiety? Cureus. (2021) 13:e15213. doi: 10.7759/cureus.15213 34178532 PMC8221086

[10] Kumar R Das A Singh V Gupta PK Bahurupi YA. Rapid survey of psychological status of health-care workers during the early outbreak of COVID-19 pandemic: a single-Centre study at a tertiary care hospital in northern India. J Med Evid. (2021) 2:213–8. doi: 10.4103/JME.JME_8_21

[11] Kumar R Beniwal K Bahurupi Y. Pandemic fatigue in nursing undergraduates: role of individual resilience and coping styles in health promotion. Front Psychol. (2022) 13:940544. doi: 10.3389/fpsyg.2022.940544 35992411 PMC9386251

[12] Dahiya H Goswami H Bhati C Yadav E Bhanupriya Tripathi D. Severe acute respiratory syndrome coronavirus 2 omicron variant and psychological distress among frontline nurses in a major COVID-19 center: implications for supporting psychological well-being. J Prim Care Spec. (2023) 4:p10–6. doi: 10.4103/jopcs.jopcs_22_22

[13] Chen J Liu X Wang D Jin Y He M Ma Y. Risk factors for depression and anxiety in healthcare workers deployed during the COVID-19 outbreak in China. Soc Psychiatry Psychiatr Epidemiol. (2021) 56:47–55. doi: 10.1007/s00127-020-01954-1 32914298 PMC7483060

[14] Hacimusalar Y Kahve AC Yasar AB Aydin MS. Anxiety and hopelessness levels in COVID-19 pandemic: a comparative study of healthcare professionals and other community sample in Turkey. J Psychiatr Res. (2020) 129:181–8. doi: 10.1016/j.jpsychires.2020.07.024 32758711 PMC7372275

[15] Al Maqbali M Al Sinani M Al-Lenjawi B. Prevalence of stress, depression, anxiety and sleep disturbance among nurses during the COVID-19 pandemic: a systematic review and meta-analysis. J Psychosom Res. (2021) 141:110343. doi: 10.1016/j.jpsychores.2020.110343 33360329 PMC7831768

[16] Peng P Chen Q Liang M Liu Y Chen S Wang Y. A network analysis of anxiety and depression symptoms among Chinese nurses in the late stage of the COVID-19 pandemic. Front Public Health. (2022) 10:996386. doi: 10.3389/fpubh.2022.996386 36408014 PMC9667894

[17] Braquehais MD Vargas-Cáceres S Gómez-Durán E Nieva G Valero S Casas M. The impact of the COVID-19 pandemic on the mental health of healthcare professionals. QJM. (2020):hcaa207. doi: 10.1093/qjmed/hcaa207 32569374 PMC7337807

[18] Ruiz-Fernández MD Ramos-Pichardo JD Ibáñez-Masero O Cabrera-Troya J Carmona-Rega MI Ortega-Galán ÁM. Compassion fatigue, burnout, compassion satisfaction and perceived stress in healthcare professionals during the COVID-19 health crisis in Spain. J Clin Nurs. (2020) 29:4321–30. doi: 10.1111/jocn.15469 32860287

[19] Labrague LJ De Los Santos JAA. COVID-19 anxiety among frontline nurses: predictive role of organisational support, personal resilience and social support. J Nurs Manag. (2020) 28:1653–61. doi: 10.1111/jonm.13121 32770780 PMC7436313

[20] Barr P. Moral distress and considering leaving in NICU nurses: direct effects and indirect effects mediated by burnout and the hospital ethical climate. Neonatology. (2020) 117:646–9. doi: 10.1159/000509311 32750693

[21] Cuschieri S. The STROBE guidelines. Saudi J Anaesth. (2019) 13:S31–4. doi: 10.4103/sja.SJA_543_18 30930717 PMC6398292

[22] Zung WW. A rating instrument for anxiety disorders. Psychosomatics. (1971) 12:371–9. doi: 10.1016/S0033-3182(71)71479-0 5172928

[23] Dunstan DA Scott N. Norms for Zung’s self-rating anxiety scale. BMC Psychiatry. (2020) 20:90. doi: 10.1186/s12888-019-2427-6 32111187 PMC7048044

[24] Zung WW. Self-rating depression scale. Arch Gen Psychiatry. (1965) 12:63–70. doi: 10.1001/archpsyc.1965.01720310065008 14221692

[25] Liu Z Qiao D Xu Y Zhao W Yang Y Wen D. The efficacy of computerized cognitive behavioral therapy for depressive and anxiety symptoms in patients with COVID-19:randomized controlled trial. J Med Internet Res. (2021) 23:e26883. doi: 10.2196/26883 33900931 PMC8128049

[26] Gül Ş Kılıç ST. Determining anxiety levels and related factors in operating room nurses during the COVID-19 pandemic: a descriptive study. J Nurs Manag. (2021) 29:1934–45. doi: 10.1111/jonm.13332 33843088 PMC8250360

[27] Santoro PE Borrelli I Gualano MR Amantea C Tumminello A Daniele A. Occupational hazards and gender differences: a narrative review. Ital J Gend-Specif Med. (2022) 8:154–62. doi: 10.1723/3927.39110

[28] Koksal E Dost B Terzi Ö Ustun YB Özdin S Bilgin S. Evaluation of depression and anxiety levels and related factors among operating theater workers during the novel coronavirus (COVID-19) pandemic. J Perianesth Nurs. (2020) 35:472–7. doi: 10.1016/j.jopan.2020.06.017 32855053 PMC7303603

[29] Mo Y Deng L Zhang L Lang Q Liao C Wang N. Work stress among Chinese nurses to support Wuhan for fighting against the COVID-19 epidemic. J Nurs Manag. (2020) 28:1002–9. doi: 10.1111/jonm.13014 32255222 PMC7262235

[30] Adams M Chase J Doyle C Mills J. Self-care planning supports clinical care: putting total care into practice. Prog Palliat Care. (2020) 28:305–7. doi: 10.1080/09699260.2020.1799815

[31] Hossain F Clatty A. Self-care strategies in response to nurses’ moral injury during COVID-19 pandemic. Nurs Ethics. (2021) 28:23–32. doi: 10.1177/0969733020961825 33124492 PMC7604672

[32] Liu N Zhang F Wei C Jia Y Shang Z Sun L. Prevalence and predictors of PTSS during COVID-19 outbreak in China hardest-hit areas: gender differences matter. Psychiatry Res. (2020) 287:112921. doi: 10.1016/j.psychres.2020.112921 32240896 PMC7102622

[33] Liu S Yang L Zhang C Xiang YT Liu Z Hu S. Online mental health services in China during the COVID-19 outbreak. Lancet Psychiatry. (2020) 7:e17–8. doi: 10.1016/S2215-0366(20)30077-8 32085841 PMC7129099

[34] Su X Guo LL. Relationship between psychological elasticity, work stress and social support of clinical female nurses. Chinese Occup Med. (2015) 42:55–8. doi: 10.11763/j.issn.2095-2619.2015.01.013

[35] Han L Wong FKY She DLM Li SY Yang YF Jiang MY. Anxiety and depression of nurses in a north west province in China during the period of novel coronavirus pneumonia outbreak. J Nurs Scholarsh. (2020) 52:564–73. doi: 10.1111/jnu.12590 32652884 PMC7405411

[36] Middeldorp CM Cath DC Van Dyck R Boomsma DI. The co-morbidity of anxiety and depression in the perspective of genetic epidemiology. A review of twin and family studies. Psychol Med. (2005) 35:611–24. doi: 10.1017/s003329170400412x 15918338

